# Editorial: Editor's challenge: Dr. Luciano Mutti - what is the true impact of ICIs on survival in the treatment of thoracic malignancies?

**DOI:** 10.3389/fonc.2023.1285031

**Published:** 2023-09-21

**Authors:** Luciano Mutti, Steven G. Gray

**Affiliations:** ^1^ Center for Biotechnology, Sbarro Institute for Cancer Research and Molecular Medicine, College of Science and Technology, Temple University, Philadelphia, PA, United States; ^2^ Department of Applied Clinical Sciences and Biotechnology, L’ Aquila University, L’ Aquila, Italy; ^3^ Department of Clinical Medicine, Trinity St James’s Cancer Institute, Trinity College Dublin, Dublin, Ireland

**Keywords:** immune check inhibitor (ICI), non-small cell lung cancer, malignancy, survival, therapy

The possibility that the human immune system can act to prevent cancer was first proposed in 1909 by Ehrlich and is often described as immune-surveillance) (1). One of the evasion mechanisms utilized by cancer cells involves the expression a series of surface regulatory markers (called checkpoint molecules) that prevent the activation of immune system responses. This has led to the development of what are called immune checkpoint inhibitors (ICIs), which are now a mainstay therapy in the treatment of many cancers including thoracic malignancies (2). Despite the development of these agents, they are not completely effective and many patients either fail to respond to ICIs (3, 4), initially respond but subsequently relapse, developing ICI resistance (5), or develop immune-related adverse events (irAEs) (6).

In this topic we asked the research community whether ICIs in their present form have a true impact on patient survival. A study by Sayer et al. examined the impact of irAEs on non-small cell lung cancer (NSCLC) patient survival, and explored if prior treatment with tyrosine receptor kinases (TKIs) played a role in patient responses, and found that indeed patients experiencing an irAE were found to have a significantly longer OS and PFS compared to patients who did not. It must be noted that this analysis is based off a single institution, and larger cross-institutional studies will be required to confirm this interesting finding. Reliable biomarkers for ICI therapy remain a core challenge for patient stratification (7), and a new candidate biomarker based on mutations in the tumor suppressor RB1 was described by Wang et al. In this study mutated RB1 was associated with poor responses to ICI outperforming both tumor mutational burden (TMB) and PD-L1 positivity. Again the study was limited to a single institution and will require further validation.

Systematic reviews are helping to assess ICI efficacy and identify new options for more effective patient stratification or treatment. Li et al. conducted a systematic review and meta-analysis on histological subtypes of NSCLC (Squamous versus non-Squamous) to see if these subtypes had any effect on efficacy or outcome. From an analysis of 40 clinical trials this study was able to conclude that both histologies benefited from ICI therapy (either as monotherapy or combinatorial therapy), and also demonstrated that under combinatorial therapy non-squamous patients derived more significant survival benefit. In a similar fashion, Yang et al. conducted a systematic review in NSCLC to assess clinicopathological and bio-molecular features that affected survival upon treatment with ICIs. From an analysis of 23 randomized controlled trials (RCTs) the authors identified wild-type EGFR, high PD-L1 expression, and high blood-based TMB (bTMB) as features associated with a significant OS benefit from ICI therapy, whereas unmutated EGFR, low PD-L1 expression, or low bTMB did not demonstrate any clear OS benefit. Subgroup analysis demonstrated that the observed OS benefit was achieved regardless of regardless of sex, age, ECOG PS, histology, smoking history, baseline brain metastasis, race, or region. These results effective confirm PD-L1 and high TMB as effective biomarkers for ICI therapy, and suggest that non-mutated EGFR be considered as part of the treatment algorithm for ICI therapy. Whilst PD-L1 positivity is normally required for ICI therapy in NSCLC, it is well established that a subset of patients with negative PD-L1 expression (PD-L1 < 1%) show objective responses to ICI (8, 9). Li et al. conducted a network meta-analysis to assess whether such patients derive better benefit from chemotherapy combined with antiangiogenic versus chemotherapy combined with ICIs as no head-to-head clinical trials comparing the two exist. Their analysis suggests that for patients with advanced NSCLC with negative PD-L1 expression, combinations of chemotherapy plus ICI provide the best benefit.

The field of immunotherapy is still young, and the data presented in this special topic suggests that whilst it remains increasingly clear that ICIs display clear survival benefit in thoracic malignancy, more research will be required to truly define their long-term impact on patient survival.

## Author contributions

LM: Writing – review & editing. SG: Writing – original draft.
